# Comparison of Microbiological, Histological, and Immunomodulatory Parameters in Response to Treatment with Either Combination Therapy with Prednisone and Metronidazole or Probiotic VSL#3 Strains in Dogs with Idiopathic Inflammatory Bowel Disease

**DOI:** 10.1371/journal.pone.0094699

**Published:** 2014-04-10

**Authors:** Giacomo Rossi, Graziano Pengo, Marco Caldin, Angela Palumbo Piccionello, Jörg M. Steiner, Noah D. Cohen, Albert E. Jergens, Jan S. Suchodolski

**Affiliations:** 1 School of Veterinary Medical Sciences, University of Camerino, Camerino, Italy; 2 Clinic “St. Antonio”, Cremona, Italy; 3 San Marco Laboratories, Padova, Italy; 4 Gastrointestinal Laboratory, Department of Small Animal Clinical Sciences, College of Veterinary Medicine and Biomedical Sciences, Texas A&M University, College Station, Texas, United States of America; 5 Department of Large Animal Clinical Sciences, College of Veterinary Medicine and Biomedical Sciences, Texas A&M University, College Station, Texas, United States of America; 6 Department of Veterinary Clinical Sciences, College of Veterinary Medicine, Iowa State University, Ames, Iowa, United States of America; INSERM, France

## Abstract

**Background:**

Idiopathic inflammatory bowel disease (IBD) is a common chronic enteropathy in dogs. There are no published studies regarding the use of probiotics in the treatment of canine IBD. The objectives were to compare responses to treatment with either combination therapy (prednisone and metronidazole) or probiotic strains (VSL#3) in dogs with IBD.

**Methodology and Principal Findings:**

Twenty pet dogs with a diagnosis of IBD, ten healthy pet dogs, and archived control intestinal tissues from three euthanized dogs were used in this open label study. Dogs with IBD were randomized to receive either probiotic (D-VSL#3, n = 10) or combination drug therapy (D-CT, n = 10). Dogs were monitored for 60 days (during treatment) and re-evaluated 30 days after completing treatment. The CIBDAI (P<0.001), duodenal histology scores (P<0.001), and CD3+ cells decreased post-treatment in both treatment groups. FoxP3+ cells (p<0.002) increased in the D-VSL#3 group after treatment but not in the D-CT group. TGF-β+ cells increased in both groups after treatment (P = 0.0043) with the magnitude of this increase being significantly greater for dogs in the D-VSL#3 group compared to the D-CT group. Changes in apical junction complex molecules occludin and claudin-2 differed depending on treatment. *Faecalibacterium* and *Turicibacter* were significantly decreased in dogs with IBD at T0, with a significant increase in *Faecalibacterium* abundance observed in the animals treated with VSL#3 strains.

**Conclusions:**

A protective effect of VSL#3 strains was observed in dogs with IBD, with a significant decrease in clinical and histological scores and a decrease in CD3+ T-cell infiltration. Protection was associated with an enhancement of regulatory T-cell markers (FoxP3+ and TGF-β+), specifically observed in the probiotic-treated group and not in animals receiving combination therapy. A normalization of dysbiosis after long-term therapy was observed in the probiotic group. Larger scale studies are warranted to evaluate the clinical efficacy of VSL#3 in canine IBD.

## Introduction

Similar to human inflammatory bowel disease (IBD), three main factors are considered to be fundamental in the pathogenesis of canine idiopathic IBD: the interactions between the mucosal immune system, host genetic susceptibility, and environmental factors (e.g., microbiota, nutrition) [1]–[3]. Experimental evidence supports a role for commensal bacteria in the pathogenesis of IBD; for example, spontaneous colitis develops in mice deficient in interleukin (IL)-2 [4] and IL-10 [5] when colonized with a complex microbiota, but not in mice raised under germ-free conditions. Recent studies suggest involvement of the intestinal microbiota in the pathogenesis of canine and feline IBD [2], [6]–[9]. Also, antibiotics such as metronidazole are useful in the treatment of IBD in humans [10] and dogs [11], and there is evidence that children with IBD respond to probiotic administration [12]. Collectively, these findings suggest that the intestinal microbiota plays a crucial role in the pathogenesis of IBD and modulation of intestinal microbiota may be beneficial in the treatment of mucosal inflammation. While probiotics are used frequently in small animal practice, there are only few published studies regarding their efficacy in dogs with chronic enteropathies. In one investigation, a probiotic cocktail was shown to reduce clinical severity in a prospective, placebo-controlled trial in dogs with food-responsive diarrhea treated with an elimination diet [13], but studies evaluating idiopathic IBD have not been reported.

VSL#3 is a high-dose, multi-strain probiotic product containing viable lyophilized bacteria consisting of 4 strains of *Lactobacillus* (*L. casei*, *L. plantarum*. *L. acidophilus*, and *L. delbrueckii* subsp. *bulgaricus*), 3 strains of *Bifidobacterium* (*B. longum*, *B. breve*, and *B. infantis*), and 1 strain of *Streptococcus sulivarius* subsp *thermophilus*. The VSL#3 strains have shown efficacy in humans for the prevention, treatment, and maintenance of remission of both pouchitis and ulcerative colitis in adults and children [12], [14], [15].

The purpose of the present study was to perform a randomized open-label trial to compare the microbiological, histological, and immunomodulatory effects between the commercial multi-strain probiotic SIVOY, a probiotic product formulated with VSL#3 strains for pets (VSL Pharmaceuticals, Inc., Gaithersburg, MD, USA) and combination therapy with prednisone and metronidazole in canine IBD.

Our results suggest a protective effect of the probiotic mixture in dogs with IBD, with a significant decrease in clinical and histological scores, and a decrease in CD3+ T-cell infiltration. Protection was associated with an enhancement of regulatory T-cell markers (FoxP3+ and TGF-β+), specifically observed in the probiotic-treated group and not in animals receiving combination therapy. The protective effect of the probiotic VSL#3 strains was also associated with normalization of dysbiosis, specifically increases in *Faecalibacterium* spp.

## Materials and Methods

### Animals

The study was approved by the Camerino University Institutional Animal Care and Use Committee protocol and all owners of the IBD dogs gave informed written consent before enrollment. Twenty pet dogs (Table 1) with a long-time diagnosis of IBD according to published criteria [16] were evaluated at the Veterinary Teaching Hospital, Camerino University, for chronic gastroenteritis. Inclusion criteria included recurrence of clinical signs and absence of any immunomodulating drug therapy (e.g., corticosteroids, metronidazole, and sulfasalazine) within a month before referral. Diagnostic criteria for IBD included: persistent (>3 weeks) gastrointestinal signs, failed responses to dietary (hydrolysate or commercial intact protein elimination diet) or symptomatic therapies (anthelminthics, antibiotics, anticholinergics, gastrointestinal protectants) alone, a thorough diagnostic evaluation with failure to document other causes for gastroenteritis, and histopathologic evidence of intestinal inflammation. The minimum diagnostic evaluation in all dogs included a complete blood count, serum biochemistry, urinalysis, direct (wet mount) and indirect (flotation) examination of feces for endoparasites, and survey abdominal radiographs. In some instances, additional tests including contrast radiography, abdominal ultrasound (performed in 16 of the 20 dogs) and measurement of serum concentrations of trypsin-like immunoreactivity and/or folate and cobalamin were performed. Additional inclusion criteria were the absence of extra-alimentary tract inflammation based on results obtained from initial diagnostic testing. Dogs with hypoproteinemia or a suspicion of intestinal lymphangiectasia were excluded from the study.

**Table 1:** Summary characteristics of enrolled dogs.

	Treatment groups		
	*VSL#3 (n = 10)*	*CT (n = 10)*	*Healthy Control (n = 10)*
**Breed**	Golden Retriever, Husky, Boxer, Rottweiler, Jack Russell Terrier, WHW Terrier, German shepherd (2), Shih Tzu, Yorkshire Terrier	Golden retriever (2), Cocker Spaniel, Boxer, Bull Terrier, Carlino, WHW Terrier, German shepherd, Shar Pei, Yorkshire Terrier	Golden Retriever, Epagneul Breton, Chow Chow, Rottweiler, Border collie, German shepherd, Bolognese, Miniature Schnauzer, Yorkshire Terrier (2)
			
			
			
			
**Sex**	m = 5, mn = 1, f = 1, fs = 3	m = 5, f = 2,fs = 3	m = 5, f = 5
**Median age (range) in years**	5.8 (2.5–11)	5.5 (1.5–9)	6.5 (1–12)
**Body weight (range) in kg**	18.9 (2–36)	18.7 (1.5–30)	20.6 (2.8–45)
**Median (range) time to remission (days)**	10.6 (5–15)	4.8 (2.5–7)	n/a

m = male, mn = neutered male, f = female, fs = spayed female; CT = combination therapy;

n/a-not applicable.

Ten pet dogs (Table 1), living in home environments and free of gastrointestinal signs for at least four months, were enrolled as control group (D–H) for comparison of fecal microbiota between healthy dogs and dogs with IBD. Control dogs were judged to be healthy based on normal results on physical examination, complete blood count, serum biochemistry, urinalysis, repeated fecal examinations, and dirofilarial antigen assay.

### Study design

The trial was a 90 day open-label evaluation to compare the effects of VSL#3 strains versus combination drug therapy on histological, microbiological, and immunological markers. Dogs were randomized into two groups using a computer-generated randomization list. The VSL#3 group (D-VSL#3; n = 10) received between 112 and 225 billion (112 to 225×10^9^) lyophilized bacteria per 10 kg daily for 60 consecutive days; the D-CT group (n = 10) received a combination protocol of metronidazole at 20 mg/Kg q12 h and prednisone at 1 mg/kg body weight/day. The clinical disease activity (CIBDAI score) was assessed at baseline (T0) and after 90 days (T1) of enrollment, which was 30 days following completion of either treatment. The CIBDAI is based on 6 criteria, each scored on a scale from 0–3: attitude/activity, appetite, vomiting, stool consistency, stool frequency, and weight loss. After summation, the total composite score is determined to be clinically insignificant (score 0–3), mild (score 4–5), moderate (score 6–8) or severe (score 9 or greater) [17].

Fecal samples were also collected at each visit then immediately stored at −80°C, until microbiota analysis. The evaluation time point 30 days post-treatment was chosen to determine whether individual dogs would relapse within 30 days following completion of either treatment regimen.

### Tissue sampling

After enrollment (time point T0) and after 90 days (T1), multiple (10–15 specimens) mucosal biopsy specimens were procured endoscopically from the small and/or large intestine of all dogs with IBD (n = 20, 10 dogs per treatment group). Fifteen dogs having predominantly upper gastrointestinal signs (i.e., vomiting, small bowel diarrhea, anorexia, and/or weight loss) underwent esophagogastroduodenoscopy, whereas upper and lower endoscopic examinations were performed in 5 dogs having mixed signs of enterocolitis (i.e., GI signs associated with tenesmus, hematochezia, mucoid feces, and/or frequent defecation). Biopsy specimens were obtained directly from mucosal lesions of increased granularity, friability, or erosions as well as areas of normal-appearing mucosa. Tissues for histopathology were placed in 10% neutral buffered formalin, then paraffin embedded and serial 3 μm thick sections were prepared. For ethical considerations, no endoscopic examinations were performed in healthy dogs. Histopathology was performed by a single pathologist, who was blinded regarding history, clinical signs, or endoscopic observations. A severity score was assigned for each dog, by using a standardized and previously described histologic grading system, based on the extent of architectural disruption and mucosal epithelial changes [17], [18], as recently been proposed by the WSAVA for diagnosis of gastrointestinal inflammation [19].

Tissues were also evaluated for expression patterns of apical junction complex (AJC) molecules in both dog groups after end of the therapy. To obtain control tissue from healthy dogs for this analysis, archived formalin-fixed and paraffin–embedded colonic tissues from three male dogs with no clinical signs of intestinal disease were retrieved from the University of Camerino Veterinary Pathology Unit archives. These samples had been obtained immediately post-mortem from dogs that were presented for euthanasia (euthanized dogs, ED) for old age (*n* = 1), nasal carcinoma (*n* = 1), or splenic haemangiosarcoma (*n* = 1). Ages ranged from 7 years to 14 years and histopathological examination of full-thickness intestinal biopsies was normal in all these ED cases.

### Immunohistochemical evaluation

Paraffin sections were rehydrated and neutralized for endogenous peroxidases with 3% hydrogen peroxide for 5 minutes followed by rinsing for 5 minutes in distilled water. For antigen retrieval, slides were incubated in three antigen retrieval solutions: citrate buffer (pH 6.0) for TGF-β, EDTA (pH 8.0) for CD3 and FoxP3, and 0.01 M Tris-EDTA buffer (pH 9.0) for claudin 2, occludin and E-cadherin in a steamer (Black & Decker, Towson, MD, USA) for 20 minutes. Non-specific binding was blocked by incubation of slides for 10 minutes with a protein-blocking agent (Protein-blocking agent, Dako, Carpinteria, CA, USA) before application of the primary antibody. Slides were incubated overnight in a moist-chamber with the following primary antibodies: monoclonal (mAb) rat anti-human CD3 (Monoclonal rat anti-human CD3 clone MCA1477, Serotec abD, Biorad Laboratories, Hercules, CA, USA) diluted 1∶50, mAb anti-mouse/rat FoxP3 antibodies (Monoclonal anti-mouse/rat FoxP3 antibodies clone FJK-16s, eBioscience, San Diego, CA, USA) diluted 1∶400, and mAb mouse anti-TGF-β (Monoclonal mouse anti-TGF-β, clone 1D11, Serotec abD, Biorad Laboratories, Hercules, CA, USA) diluted 1∶25 [19], [20]. Polyclonal rabbit anti-claudin-2 (Polyclonal rabbit anti-claudin-2 (PAD: MH44), Invitrogen Ltd., Paisley, UK) and anti-occludin (anti-occludin PAD: Z-T22, Invitrogen Ltd., Paisley, UK) antibodies and monoclonal mouse anti-E-cadherin IgG2α (Monoclonal mouse anti-E-cadherin IgG2α (clone: 36), BD Biosciences, Oxford, UK) were used as described previously [21].

The immunoreaction with streptavidin–immunoperoxidase (Streptavidin–immunoperoxidase, Black & Decker, Towson, MD, USA) was visualized with 3,3′-diaminobenzidine substrate (3,3′-diaminobenzidine substrate, Vector, Burlingame, UK). Tissues were counterstained with Mayer's hematoxylin. For negative immunohistochemical controls the primary antibodies were omitted. Sections of canine spleen and tonsil served as positive control tissues for CD3 and FoxP3 cell staining and sections of canine placenta for that of TGF-β expression. Positive control tissues for claudin/occludin and E-cadherin staining consisted of canine lung and kidney sections, respectively.

For scoring of intestinal CD3+ T-lymphocytes, FoxP3+ cells, and TGF-β+ cells, these cells were quantified in select compartments of the GI tract (small intestine: villi, basal crypt area, villus-crypt junction; large intestine: apical crypt area, basal crypt area). All cellular types were evaluated using a light microscope (Carl Zeiss, Jena, Germany), a×40 objective, a×10 eyepiece, and a square eyepiece graticule (10×10 squares, having a total area of 62,500 μm^2^). Ten appropriate fields were chosen for each compartment and arithmetic means were calculated for each intestinal region. Results were expressed as IHC positive cells per 62,500 μm^2^. For all parameters, cells on the margins of the tissue sections were not considered for evaluation to avoid inflation of positive cell numbers.

For the evaluation of different lymphocytes subsets in the same histological sections, consecutive 3-μm-thick bioptic cross sections were cut. Sections were placed consecutively on each of eight separate slides, after which the ninth section was placed on the first slide, next to the first section, continuing for 48 sections. A single slide, upon which were six bioptic cross sections from each dog, was analyzed for any given immunostain. Numbers of CD3+ T-lymphocytes, FoxP3+ cells, and TGF-β+ cells, were quantified by using an image-analysis system consisting of a light microscope (Carl Zeiss, Jena, Germany) attached to a Javelin JE3462 high-resolution camera and a personal computer equipped with a Coreco-Oculus OC-TCX frame grabber and high-resolution monitor. Computerized color-image analysis was performed by using Image-Pro Plus software (Media Cybernetics). The area of each biopsy in all six cross sections in every dog was recorded, as was the total number of T-lymphocytes determined by immunostaining as previously described. For each dog, the total bioptic area was calculated as the sum of the areas of all fields in all six bioptic cross sections on one slide. CD3+ T-lymphocytes, FoxP3+ cells, and TGF-β+ cells were counted per section, and stained cell densities were expressed as the number of lymphocytes/cells per square millimeter of analyzed bioptic area [22].

To assess AJC expression (claudin-2, occludin, and E-cadherin) in biopsies sampled after treatment in both groups (at T1 for D-VSL#3 and D-CT), and to compare data to the AJC expression in non-IBD control dogs (ED group), stained tissue sections were evaluated at ×200 and ×630 (oil immersion) magnification to identify areas of consistent staining and acceptable orientation. Immunostaining was evaluated along the length of multiple enteric/colonic crypts and in areas of intact luminal epithelium. Stain intensity was subjectively graded as absent (−), weak (+), moderate (++), or strong (+++), and the localization and distribution of chromogen were noted. For evaluation, intestinal epithelium was divided into luminal, proximal, and distal gland/crypt regions, and the intercellular junction was divided into apical and basolateral compartments. Finally, the scoring of intestinal AJC molecules expression was calculated as previously described for CD3+ T-cells, FoxP3+ cells, and TGF-β+ cells. The AJC molecules were assessd only at T1 (following treatment intervention), because at T0 all dogs had endoscopically visible lesions of intestinal inflammation including erosions, friability, and increased mucosal granularity. Also, dogs had histopathologic lesions of intestinal inflammation of varying severity. Intestinal inflammation was associated with different degrees of epithelial infiltration by lymphocytes (i.e., intraepithelial lymphocytes) in all dogs of both groups. In these instances, it was not considered useful to evaluate AJCs as they were assumed to be altered, but instead AJC molecules were evaluated at T1 when the previously observed endoscopic lesions of inflammation had resolved.

### Plasma citrulline

Plasma concentrations of citrulline were measured in the D-VSL#3 treated group only. Plasma samples were taken at baseline (T0) and after 90 days (T1) and stored at −80°C until evaluation. Samples were precipitated with organic solvents and quantified by MS/MS mass spectrometer equipped with electrospray ionization (ESI) interface in positive ion mode (Waters TQ Detector, Water Corp., Milford, MA, USA). All assays were performed in duplicate fashion. All data collected in centroid mode were processed using commercial software (MassLynx 4.1 software, Water Corp., Milford, Ma, USA).

### Microbiota analysis

Fresh naturally voided samples were collected from all 20 diseased dogs (at T0 and T1) and 10 healthy dogs (one time point), flash-frozen in liquid nitrogen and stored at −80°C. DNA was extracted using a bead-beating method (PowerSoil DNA Isolation Kit, MoBio Laboratories, Carlsbad, CA) according to the manufacturer's protocol. Selected bacterial groups within the fecal microbiota were analyzed by quantitative PCR (qPCR) assays as described previously for canine fecal samples (Table 2) [9], [23]. Amplified DNA from each bacterial group was normalized for total amplified bacterial DNA (log_10_ amplified DNA for each bacterial group divided by log_10_ of amplified bacterial DNA) as described previously [23].

**Table 2:** Oligonucleotides primers/probes used in this study.

qPCR primers/probe	Sequence (5′-3′)	Target	Annealing (°C)	Reference
Forward	CCGGAWTYATTGGGTTTAAAGGG	Bacteroidetes	60	[45]
Reverse	GGTAAGGTTCCTCGCGTA			
Forward	GAAGGCGGCCTACTGGGCAC	*Faecalibacterium*	60	[47]
Reverse	GTGCAGGCGAGTTGCAGCCT			
Forward	ACTGAGAGGTTGAACGGCCA	Family Ruminococcaceae	59	[47]
Reverse	CCTTTACACCCAGTAAWTCCGGA			
Forward	CGCATAACGTTGAAAGATGG			
Reverse	CCTTGGTAGGCCGTTACCC	*C. perfringens* 16S	58	[48]
Probe	TCATCATTCAACCAAAGGAGCAATCC			
Forward	KGGGCTCAACMCMGTATTGCGT	Fusobacteria	51	[9]
Reverse	TCGCGTTAGCTTGGGCGCTG			
Forward	TCTGATGTGAAAGGCTGGGGCTTA	*Blautia*	56	[9]
Reverse	GGCTTAGCCACCCGACACCTA			
Forward	CCTACGGGAGGCAGCAGT	Universal Bacteria	59	[49]
Reverse	ATTACCGCGGCTGCTGG			
Forward	CAGACGGGGACAACGATTGGA	*Turicibacter*	63	[9]
Reverse	TACGCATCGTCGCCTTGGTA			
Forward	TCGCGTCCGGTGTGAAAG	*Bifidobacterium*	60	[50]
Reverse	CCACATCCAGCATCCAC			
Forward	CCCTTATTGTTAGTTGCCATCATT	*Enterococcus*	61	[46]
Reverse	ACTCGTTGTACTTCCCATTGT			
Forward	AGCAGTAGGGAATCTTCCA^a^	*Lactobacillus*	58	[46]
Reverse	CACCGCTACACATGGAG^b^			
Forward	TTATTTGAAAGGGGCAATTGCT	*Streptococcus*	54	[51]
Reverse	GTGAACTTTCCACTCTCACAC			

a Originally described by [52].

b Originally described by [53].

### Data analysis (statistics)

To evaluate differences at baseline as well as post-treatment between both treatment groups, a combined statistical analysis model was used. This model takes into account differences between the treatment groups at T0 as well as post-treatment at T1. The effects of time (i.e., T0 or T1), treatment, and their 2-way interaction on the various outcome parameters were measured (viz., histology, CIBDAI, TGF-β+, CD3+, and FoxP3+ cells). Dog was modeled as a random effect to account for repeated measures (before and after treatment) for individual dogs; time, treatment, and their 2-way interaction were modeled as fixed, categorical variables. Datasets for TGF-β and FoxP3+ were log_10_-transformed to meet distributional assumptions underlying the statistical modeling. Confidence intervals (CIs) were estimated using maximum likelihood methods. The correlation structure for mixed-effects modeling was that of compound symmetry. Model fit was assessed visually by examining plots of standardized residuals versus fitted values, and by examining the AIC and BIC values for models. A significance level of P<0.05 was used for all analyses (S-PLUS, Version 8.2, TIBCO, Inc., Palo Alto, CA, USA). Changes in plasma citrulline concentrations were compared in the D-VSL#3 group between T0 and T1 using a Wilcoxon matched pairs test. The expression of AJC molecules expression (claudin-2, occludin, and E-cadherin) were compared at T1 between the dogs in the D-VSL#3, the D-CT, and the ED group using a Kruskal-Wallis-Test. The microbiota data obtained by qPCR were compared between healthy dogs and both treatment groups at T0 using an ANOVA or Kruskal-Wallis test where appropriate after evaluating for normal distribution using the Kolmogorov–Smirnov test. Changes in bacterial groups between T0 and T1 were compared using Wilcoxon matched pairs tests. Resulting P-values were corrected for multiple comparisons using the false discovery rate as described by Benjamini & Hochberg, and a P<0.05 was considered significant [9].

## Results

**Figure 1:**
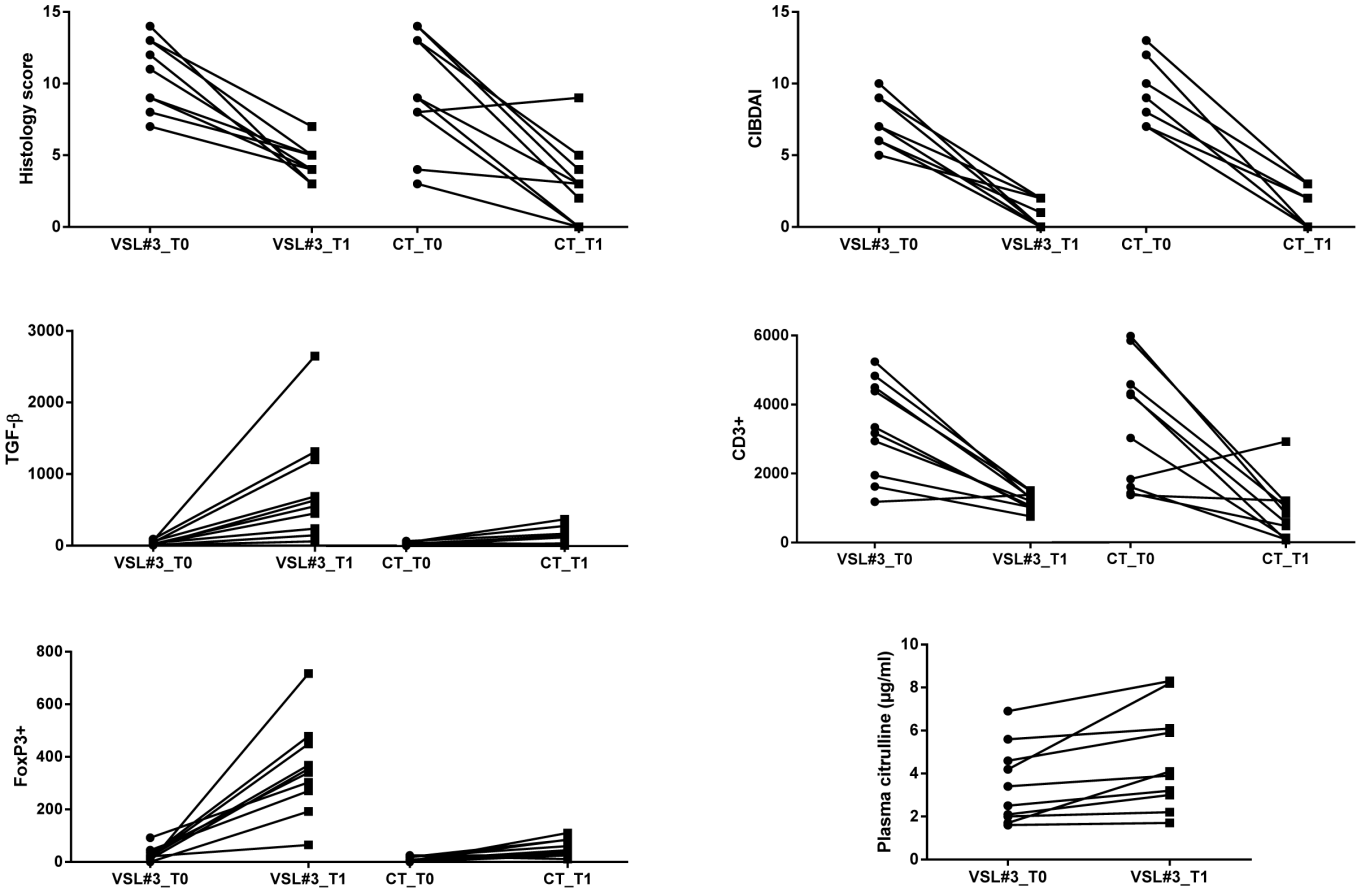
Results for histology scores, CIBDAI, CD3+ cells, FoxP3+ cells, TGF-β cells, and plasma citrulline concentrations. Significant differences between baseline (T0) and 30 days after the end of therapy (T1) were observed for all parameters in both treatment groups except the expression of FoxP3+ T-cells in the CT group (P = 0.3296). While TGF-β increased significantly in both treatment groups, the magnitude of the increase was significantly higher in dogs treated with VSL#3 (P = 0.0008). Data for CD3+ cells, FoxP3+ cells, TGF-β cells expressed as cells per 62,500 μm^2^.

Table 1 summarizes the signalment of the dogs enrolled into the study. No significant differences for age, sex, or body weights were identified (P>0.05 for each) between the dog groups. Table 3 and Figure 1 summarize the changes in histology scores, CIBDAI, and TGF-β+, FoxP3+, and CD3+ T-cell expression in both treatment groups.

**Table 3:** Summary statistics for evaluated markers.

	*VSL#3*		*CT*		*ED*	
	*T0*	*T1*	*T0*	*T1*	*-*	
**Histology score**	11.5 (7–14)	4 (3–7)	9 (3–14)	3 (0–9)	—	P<0.0001*
**CIBDAI score**	7 (5–10)	0 (0–2)	9 (7–13)	0 (0–3)	—	P<0.0001*
**CD3+ cells** †	3318 (±447.1)	1204 (±240.4)	3427 (±1813)	845 (±849)	—	P<0.0001*
**FoxP3+ cells** †	26.9 (±26.9)	353.6 (±175.1)	11.1 (±9.5)	51.5 (±32.2)	—	P = 0.0001**
**TGF-β+ cells** †	35.4 (±30.3)	791.8 (±771.9)	32.6 (±21.8)	136.7 (±122)	—	P = 0.0043*
**Citrulline (μg/ml)**	3.46 (±1.82)	4.66 (±2.34)	—	—	—	P = 0.0113
**E-caderin** †	—	4767 (±2288)	—	4735 (±1319)	4877 (±971)	P = 0.9467
**Occludin** †	—	4523 (±1366)	—	814 (±387)	6511 (±1239)	P<0.0001
**Claudin-2 (SI)** †	—	4274 (±1201)	—	4421 (±1293)	4994 (±1183)	P = 0.7944
**Claudin-2 (LI)** †	—	525 (±264)	—	5771 (±1588)	680 (±305)	P<0.0001

Numerical data are expressed as median (range) for histology and CIBDAI and as mean (± SD) for remaining data.

*significant differences between T0 and T1 in both treatment groups.

**significant differences between T0 and T1 for the VSL#3 group only.

† cells per 62,500 μm^2^.

### Histology scores

**Figure 2:**
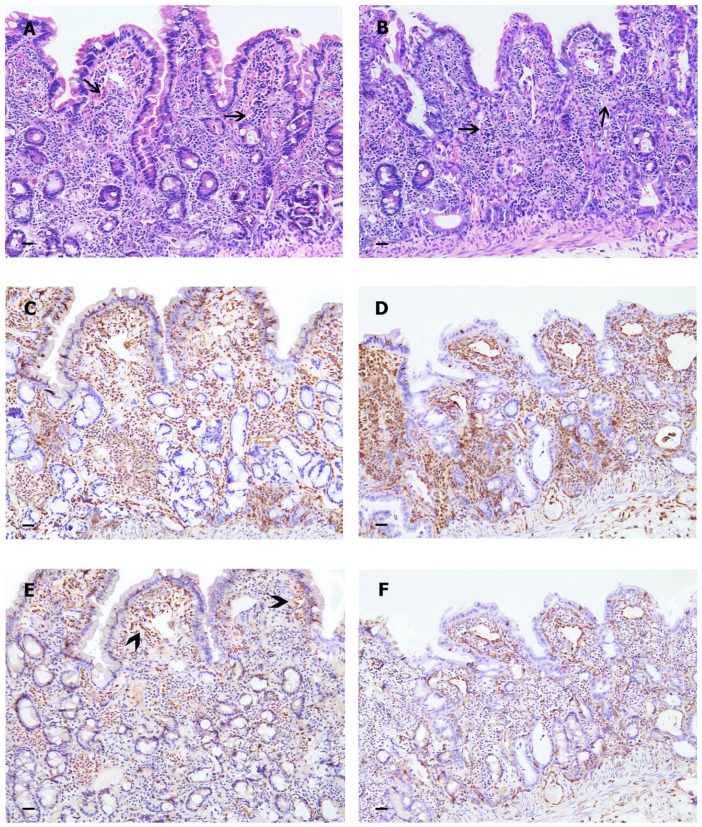
Histology of intestinal mucosa of dogs with IBD after treatment with VSL#3 (A, C, E) and CT (B, D, F). A residual inflammatory infiltrate with lymphocytic-plasmacytic cells (arrows) is evident after the therapy in both samples (H&E, 40X). In both treatment groups ssimilar patterns of mucosal infiltrations with CD3+ T-lymphocytes are evident (C and D). Infiltration with Fox-P3+ cells are proportionally increased in a sample belonging to a VSL#3 treated dog (E) compared to the sample from CT treated dog (F). Note the particular Fox-P3+ T-cells concentrations at the apical portion of villi in the VSL#3 treated dog (E) (arrow-heads) (IHC, ABC method, Harris haematoxylin nuclear counterstain, 40X).

Although there was a residual inflammatory infiltrate present (Figure 2), histology scores were significantly (P<0.0001) reduced at T1 relative to T0 in both treatment groups (Table 3 and Figure 1). There were no significant differences in the magnitude of this reduction between treatments (P = 0.1452).

### CIBDAI

Despite computer randomization, the severity of clinical signs, as judged by the CIBDAI score, was significantly higher at baseline in the D-CT group (median 9, range 7–13) compared to the D-VSL#3 group (median 7, range 5–10; P<0.0001). This was, in part, related to the fact that the all 3 dogs with severe disease were randomly allocated to the D-CT group. Another explanation was the presence of active (versus quiescent) clinical disease at presentation in some dogs. Both groups, however, had overall moderate-to-severe median disease activity at presentation. Clinical scores decreased significantly in both treatment groups over time (P<0.0001). As reported by the owners, recovery was more rapid in the D-CT group compared to the D-VSL#3 group (P = 0.0011). The median time of clinical remission of the main clinical sign (i.e., diarrhea or vomiting) of the dogs in the D-CT group was 4.8 days (range, 2.5 to 7.0 days); while in D-VSL#3 group an improvement was observed in a median of 10.6 days (range, 5.0 to 15.0 days).

### TGF-β+

While the TGF-β expression increased significantly in both treatment groups between T0 and T1 (P = 0.0043), the magnitude of this increase was significantly greater for dogs in the D-VSL#3 group than those for the dogs in the D-CT group at Time T1 (P = 0.0008) without any obvious preferential localization throughout the small or large intestine (Figure 1).

### CD3+ T-cells

The number of CD3+ lymphocytes was increased in dogs with IBD in both treatment groups at T0 (before treatment), with small or large intestinal involvement depending of intestinal tract involved in the inflammatory process. CD3+ T-cells were significantly (P<0.0001) reduced at T1 relative to T0 in both treatment groups (Figure 1 and 2), and there were no significant differences in the magnitude of this reduction between both treatments (P = 0.7527).

### FoxP3+ cells

At T0, there were no significant differences in the number of cells between the two treatment groups (Figure 1). No significant increase in FoxP3+ cells was observed in the D-CT group (P = 0.3296). However, a significant increase in FoxP3+ cells between T0 and T1 (P = 0.0001) was observed in the D-VSL#3 group.

### Expression of AJC proteins

**Figure 3:**
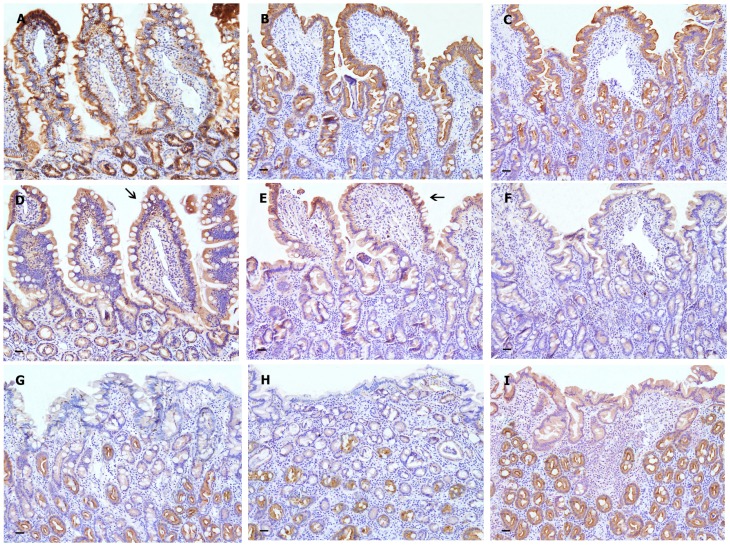
Expression of AJC proteins in the intestinal mucosa of control dogs (ED group) (A, D, G) and dogs treated with VSL#3 (B, E, H) or CT (C, F, I). No discernible differences in the distribution or staining intensity of E-cadherin are observed between normal mucosa (A) and IBD samples (B and C); the overall intensity of E-cadherin staining decreased from the luminal epithelium to the distal crypts. Occludin-specific labelling is most intense at the epithelial cell AJC (arrows) of the luminal epithelium covering the apical portion of villi in ED (D) and VSL#3 (E); a weak to absent expression is observed in the luminal epithelium and in some intestinal glands of the small intestine of the CT sample (F). In colonic samples belonging to ED (G) and VSL#3 (H) groups, claudin-2 is readily detectable only in the colonic crypt epithelium, decreasing in intensity from the distal to the proximal crypt and becoming barely detectable at the luminal surface of the colon. In contrast, claudin-2 expression is increased in the proximal crypt and luminal epithelium of all samples from CT dogs (I).

**Figure 4:**
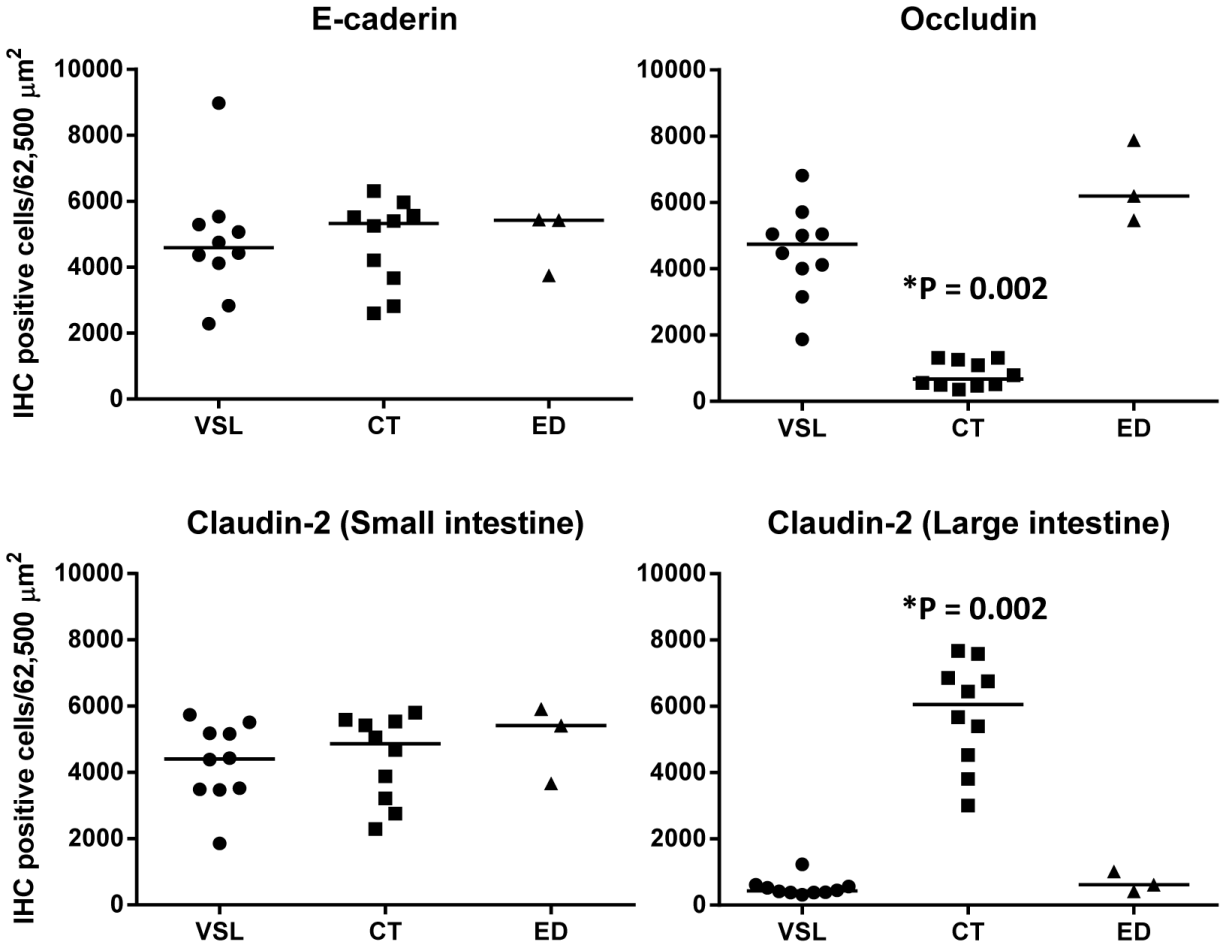
Expression of AJC proteins. Mucosal biopsies were evaluated after the end of treatment (T1) either with the probiotic (VSL) or combination drug therapy (CT), and compared to archived mucosal samples from dogs euthanized for non-gastrointestinal disorders (ED). (*significantly different to the other 2 groups; line denotes median).

Mucosal biopsies were evaluated in both treatment groups at T1 (Figure 3 and 4). Additionally, samples from 3 ED dogs were utilized as controls. Occludin was significantly lower in the D-CT group (P<0.0001) compared to the D-VSL#3 and ED groups. In contrast, Claudin-2 in the large intestine was significantly higher in the D-CT group (P<0.0001; Table 3, Figure 4) compared to the D-VSL#3 and ED groups. No significant differences were observed for the other AJC proteins.

Stain intensity was subjectively graded as absent (−), weak (+), moderate (++), or strong (+++), and the localization and distribution of chromogen was noted. Occludin-specific labeling was most intense at the epithelial cell AJC (Fig. 3), with fainter labeling observed along the basolateral membranes. This staining appeared to be uniformly expressed throughout the epithelium of both ED and D-VSL#3 groups. On the contrary, weak to absent expression was observed in the luminal epithelium and in the small intestinal glands of some dogs in the D-CT group. No discernible difference in the distribution or staining intensity of E-cadherin was observed between normal and affected dogs; as, the overall intensity of E-cadherin expression decreased from the luminal epithelium to the distal crypts. At the luminal epithelium, labeling was uniform along the length of the intercellular junction, while the expression was becoming polarized toward the AJC in the distal glands/crypts. E-cadherin-specific labeling was restricted to the AJC and basolateral membranes of intestinal epithelial cells. Moreover, there was little evidence of specific labeling outside the epithelium. In ED and D-VSL#3 groups, claudin-2 was readily detectable in the duodenal epithelium and glands and in colonic crypt epithelium. Immunostaining decreased in intensity from the distal to the proximal crypt and was minimally detectable at the luminal surface of the colon. Claudin-2-specific labeling was largely restricted to the epithelial cell AJC, with some punctate basolateral labeling noted. However, claudin-2 expression was increased in the proximal crypt and luminal epithelium in all CT dogs.

### Citrulline

Plasma citrulline concentrations increased significantly in dogs in the D-VSL#3 group between T0 and T1 (P = 0.0113; Figure 1 and Table 3).

### Microbiota analysis

**Figure 5:**
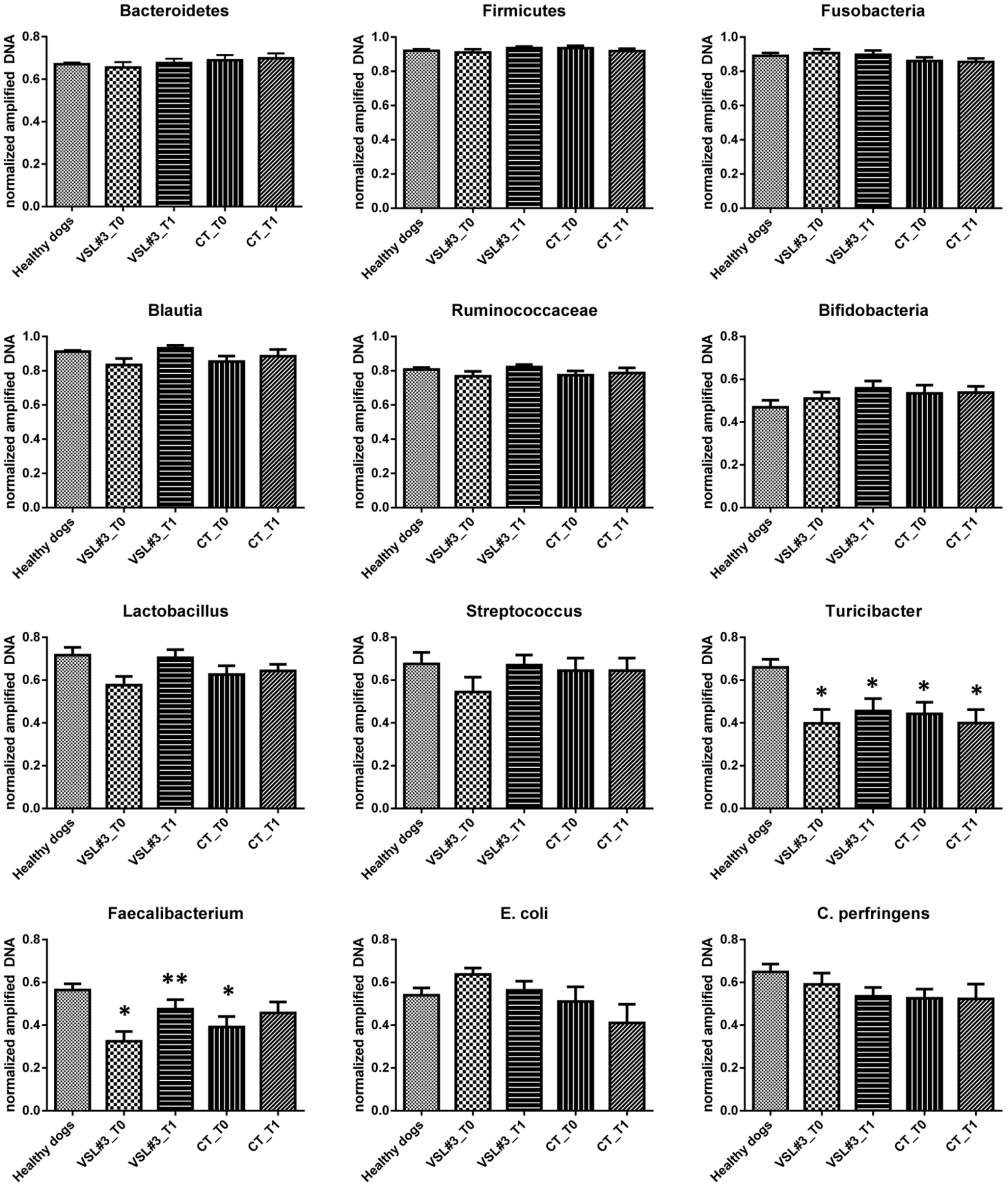
Results of quantitative PCR assays for selected bacterial groups. Dogs with IBD (in both treatment groups) had significantly decreased abundance of *Faecalibacterium* spp. (p = 0.008) and *Turicibacter* spp. (p = 0.0078) compared to the healthy dogs. *Faecalibacterium* spp. increased significantly in the VSL#3 treated dogs at T1 but not in the CT group. (*significantly different compared to healthy dogs; **significantly different after treatment compared to pre-treatment).

The qPCR results (Figure 5) showed that at T0 dogs with IBD (in both treatment groups) had significantly decreased abundance of *Faecalibacterium* spp. (p = 0.008) and *Turicibacter* spp. (p = 0.0078) when compared to healthy dogs. No other bacterial groups evaluated were significantly different compared to the healthy dogs. The qPCR analysis, revealed that the abundance of *Faecalibacterium* spp. increased significantly in the D-VSL#3 group (T1 vs T0; p = 0.03) but not in the D-CT group (T1. vs. T0; p = 0.46). No significant changes were observed for any other bacterial groups in response to treatment.

## Discussion

In this study, 20 dogs with long standing IBD were randomized to receive either a probiotic containing VSL#3 strains (SIVOY) or a combination therapy of prednisone and metronidazole. Using a statistical analysis that takes into account differences between the treatment groups at enrollment (T0) as well as post-treatment (T1), we observed differences in some of the evaluated variables depending on the treatment regimen. Histology scores, CIBDAI, and infiltration with mucosal CD3+ T-cells decreased significantly in both treatment groups, and there was no significant effect between the two treatments. FoxP3+ T-cells increased in dogs treated with VSL#3 but not in the D-CT group. While TGF-β+ cells increased significantly in both treatment groups, the magnitude of the increase was significantly greater in dogs treated with VSL#3. The expression of occludin and claudin-2 was also significantly different between dogs treated with probiotic VSL#3 compared to combination therapy.

Although the etiology of canine IBD is poorly understood, there is evidence from clinical observations, studies in humans, and animal models to incriminate the intestinal microbiota as one factor influencing aberrant host responses. Evidence for the role of enteric microbiota in the pathogenesis of IBD in humans is supported by clinical responses to fecal stream diversion in patients with Crohn's disease (CD) and antimicrobial therapy in both CD and ulcerative colitis (UC) patients [3], [10]. Furthermore, genetic mutations in NOD2/CARD15 and TLR-4 (Toll-like-receptor-4) in IBD patients make them less able to respond to bacterial components, resulting in defective innate immune responses to enteric microbiota [24]. Dietary factors also appear to play a role in mediating mucosal inflammation in dogs based on the beneficial clinical response to elimination or “hypoallergenic” diets in many of these animals [16]. All 20 patients enrolled in this study were diagnosed as having long-standing idiopathic IBD, and in the past had undergone unsuccessful dietary trials (e.g., elimination diets to exclude adverse food events). During the study period, all dogs remained on their pre-trial diets, and no dietary changes were performed as part of the here presented study. These diets were similar in nutritional composition across both treatment groups. In the D-VSL#3 group, 4 dogs were on an Adult Dry Maintenance Diet, 4 dogs were on a novel protein diet, and 2 dogs were on an elimination diet. The dogs in the D-CT group had a similar diet distribution, with 4 dogs receiving an Adult Dry Maintenance Diet, 5 dogs receiving a novel protein diet, and 1 dog receiving an elimination diet. Therefore, it is unlikely that the diets were a significant confounding factor in this study since they were broadly similar in both treatment groups.

Probiotic therapy is becoming increasingly popular in veterinary medicine, and has been recommended for the treatment or prevention of a variety of gastrointestinal disorders. However, few objective studies attesting clinical efficacy of probiotics for gastroenteritis are available. The administration of probiotics to dogs with IBD represents warrants further investigation. It has been demonstrated that colitis in both humans and mice is associated with increased levels of cytokines such as TNF-α, IL-6, IL-12p70 and IL-23 [25], [26]. Thus, a proper selection of probiotic strains for the treatment of IBD is crucial and should be based on their potential ability to induce an anti-inflammatory pattern of cytokines (IL- 10^high^, TGF-β^high^, IL-12p70^low^, IL-23^low^, TNF-α^low^) and attenuate intestinal inflammation. Apart from their immunomodulatory effects, it has been suggested that probiotics have an effect on the gut microbiome by their antimicrobial activities directed toward intestinal pathogens [3]. In humans, VSL#3 showed efficacy for maintenance of remission of ulcerative colitis [14]. To our knowledge, this is the first study to investigate the microbiological, histological, and immunomodulatory effects of VSL#3 in dogs with IBD and to compare these effects to a commonly used combination therapy with prednisone and metronidazole.

Based on qPCR analysis, only the bacterial genera *Faecalibacterium* and *Turicibacter* were found to be significantly decreased in dogs with IBD at baseline relative to healthy dogs. These results are consistent with recent findings [9], where *Faecalibacterium* was also the predominant bacterial group decreased in fecal samples of dogs with IBD. *Faecalibacterium prausnitzii* is also consistently decreased in human IBD patients and considered an important bacterial group for maintaining microbial homeostasis [27]. A suggested direct immunomodulatory mechanism of action of *F. prausnitzii* is the secretion of metabolites with anti-inflammatory effects, due to blocking NF-κB activation and IL-8 production [27]. In contrast to previous findings, Fusobacteria were not significantly different in dogs with IBD relative to healthy dogs in the current study [9]. Neither treatment with VSL#3 nor with conventional therapy led to major changes in the overall microbial abundance of bacterial phyla (Bacteroidetes, Firmicutes, Fusobacteria) as assessed 30 days following discontinuation of treatment (at T1). Figure 5 illustrates that there were no significant luminal increases in the administered probiotic genera (i.e., *Bifidobacterium, Lactobacillus*, and *Streptococcus*) in dogs receiving VSL#3. This is in line with some studies demonstrating that the administration of probiotics do appear to have only minor and transient detectable effects on fecal microbial communities as assessed by qPCR assays or sequencing of 16S rRNA genes [23], [28], [29]. In the VSL#3 group, however, *Faecalibacterium* spp. increased significantly after treatment, although a trend for an increase in this bacterial group was also observed in the CT group. These results are in line with a previous study, in which *Faecalibacterium* increased after 4 months of conventional treatment in dogs with IBD and this increase correlated with the improvement in clinical disease activity [9]. This would suggest that the significant increase in fecal *Faecalibacterium* is not necessarily specific for the probiotic treatment, but may be a general indicator for normalization of fecal dysbiosis after long-term therapy. The *Faecalibacterium–Subdoligranulum* group is a major bacterial group in the canine gastrointestinal tract, comprising 16% of total bacterial counts in feces of healthy dogs and is believed to be of importance in canine gastrointestinal health [30]. Therefore, more-in depth studies evaluating the functional properties of canine *Faecalibacterium* strains are warranted. Some limitation of the microbiota analysis performed in this study need to be noted. Analyzing the fecal microbiota using sequencing of 16S rRNA genes may have revealed potential changes either in microbial diversity indices or in bacterial groups that were not covered by our qPCR assays. For technical reasons, a sequencing approach was not possible in this study. However, we have utilized qPCR assays targeting the microbiota on various phylogenetic levels and also targeting bacterial groups that are major bacterial groups in the canine intestine and that have been shown to be important in canine IBD [9]. Furthermore, in the current study, only fecal samples were analyzed, and the potential impact of treatment on the composition of the small intestinal mucosa-associated microbiota may have been missed. Previous studies have revealed that dogs with IBD have significant differences in small intestinal microbiota compared to controls, and future studies should evaluate the effect of probiotics on the small intestinal microbiota of these dogs [8]. Also, in this study we assessed the fecal microbiota 30 days after the discontinuation of therapy, and it is possible that a transient change in the fecal microbiota during the administration period may have remained undetected and/or changed during the 30 days post-treatment.

It has been speculated that IBD is associated with a loss of intestinal barrier function, as multiple genes encoding for proteins responsible for maintenance of intestinal barrier function (i.e., those encoding for claudin-8, metallothionein, and matrix metalloproteinases) were down-regulated in dogs with IBD in a previous study [31]. The observation that the expression and distribution of occludin and claudin-2 in the large intestine were not significantly different between dogs treated with VSL#3 and the non-IBD control dogs (ED group), but were significantly different compared to the D-CT group, suggests potential effects of VSL#3 on intestinal barrier function, warranting further studies [32]. Similar changes in the distribution of claudin-2 expression have been observed in humans with active UC, where claudin-2 was detected at the surface epithelium [33]. Similarly, down regulation of occludin has been observed in the intestinal mucosa of patients with both UC and CD [34]. Here we compared the expression patterns of AJC proteins between healthy dogs (euthanized dogs; group ED) and dogs with IBD after the two different types of treatment (VSL # 3 or CT treated dogs). The expression pattern of AJC proteins in the ED group was similar to that described by Ohta *et al.* in healthy dogs [35]. In contrast, based on our results it seems that dogs in the CT-group had a greater deviation from the physiological conditions in expression of Claudin-2 in the colon. This particular expression pattern resembles that observed in samples from the colon of dogs with colitis [21]. While we cannot conclusively state that there was an improvement in the expression pattern after probiotic treatment, as samples were not evaluated at T0, we speculate that the expression pattern of AJC proteins in dogs treated with VSL#3 appears to resembles more the physiological state as observed in healthy dogs [35]. Future studies are warranted to confirm this observation. At this point it remains also unclear why claudin-2 is increased in the large intestine of dogs treated with drug therapy, and further work is needed to elucidate the mechanism behind this increased expression of claudin-2.

Dogs treated with VSL#3 showed significantly increased plasma citrulline concentrations 30 days after end of administration, suggesting restitution of the mucosal barrier. Plasma citrulline concentrations are a marker of global enterocyte mass in humans, rodents, and pigs [36], and have recently been shown to reflect intestinal mucosal recovery in response to severe injury in dogs [37]. Unfortunately, we were able to statistically evaluate the blood levels of citrulline only in the D-VSL#3 group, as plasma citrulline concentrations were not available for all dogs in the D-CT group. Because of the small samples size in the D-CT group, we decided not to perform any statistical analysis to compare plasma citrulline concentrations between treatments. Therefore, it is currently unknown whether the observed increase in plasma citrulline concentrations was specific for the treatment with VSL#3 strains, or would also be present in dogs treated with conventional therapy.

The immunohistochemical results showed cross-reactivity for canine tissues of all antibodies used in this study. This is in line with results from previous studies which have shown that these antibodies are useful for immunohistochemical assessment of canine tissues. In particular, cross-reactivity of the rat anti-human CD3 antigen, clone MCA1477, for canine CD3 positive T-lymphocytes has been shown previously on gastric tissue of dogs [20]. Cross-reactivity of the clone FJK-16s used to stain canine FoxP3-lymphocytes has been reported in another study [38]. Similarly, other authors have successfully used the monoclonal antibody against TGF-β positive dog lymphocytes (clone 1D11) [39]. Finally, the specificities of the antibodies used for canine AJC proteins (i.e., pAb anti-claudin-2 (PAD: MH44), anti-occludin (PAD: Z-T22), and mAb anti-E-cadherin (IgG2α, clone: 36) were, similarly to our study, also reported on sections of intestinal tissue in dogs with IBD [21].

The evaluation of immunomorphological variables suggests a potential anti-inflammatory effect of VSL#3 strains, as decreased mucosal CD3+ T-lymphocytes, and increased FoxP3+ and TGF-β+ positive cells were observed 30 days after the end of administration. Immunohistochemistry results showed a difference in the predominant immunophenotype of infiltrating cells in intestinal lamina propria of biopsies from VSL#3 treated dogs. More specifically, the VSL#3 treated dogs showed increases in CD3+/FoxP3+ cells (Figure 2) in the intestinal mucosa, while dogs treated with prednisone and metronidazole displayed an overall decrease in all inflammatory cell populations that was accompanied by a decrease of FoxP3+ lymphocytes and TGF-β expressing cells (Figure 2). These findings are consistent with a previous study in a mouse model, where VSL#3 also led to increased FoxP3+ expressing T-cells in intestinal lymphoid follicles [40]. In clinical studies with human IBD patients as well as studies on rodent models of IBD, VSL#3 has shown various other anti-inflammatory mechanisms. For example, VSL#3 was shown to induce heat-shock-proteins in intestinal epithelial cells (IEC) [41] or enhance proliferation of IL-10-dependent TGF-β-bearing regulatory T-cells in Th1-dependent murine colitis [42]. These variables have not been examined in the current study, and it would be useful to evaluate these markers in future clinical studies. Furthermore, qPCR quantification of both pro-inflammatory (i.e., TNF-α, IL1-β, IL-8) as well as regulatory genes (FoxP3, IL-10) would have been useful to perform since canine probes have already been published [43] and these studies showed increases in IL-8 in colorectal inflammation [44].

As limitations to this study it should be noted that only a small number of dogs was evaluated, and the power to detect differences in some of the evaluated variables may have been insufficient to detect differences between treatment groups. Furthermore, this was an open-label study and no placebo group was included. Ideally, the clinical effect of the treatment with probiotic strains should be evaluated in a double-blinded placebo controlled trial and compared to a non-treated group. However, in the case of chronic IBD, it is difficult to enroll a non-treated group as these dogs show chronic signs of disease, and therefore we chose in this study to compare the effects of VSL#3 strains to the commonly used combination therapy with prednisone and metronidazole. Our study results suggest that probiotic treatment induces differential anti-inflammatory immune responses when compared to routine combination therapy as evidenced by significant increases in FoxP3+ cells and a significantly larger increase in TGF-β. The findings lay the foundation for future larger scale placebo controlled clinical studies to evaluate clinical benefits of probiotic VSL#3 strains in the treatment of dogs with IBD.
